# Zinc Metalloprotease ProA from *Legionella pneumophila* Inhibits the Pro-Inflammatory Host Response by Degradation of Bacterial Flagellin

**DOI:** 10.3390/biom12050624

**Published:** 2022-04-22

**Authors:** Lina Scheithauer, Stefanie Thiem, Can M. Ünal, Ansgar Dellmann, Michael Steinert

**Affiliations:** 1 Institut für Mikrobiologie, Technische Universität Braunschweig, Spielmannstr. 7, 38106 Braunschweig, Germany; l.scheithauer@tu-bs.de (L.S.); stefanie.thiem@mail.de (S.T.); c.uenal@tu-bs.de (C.M.Ü.); 2 Institut für Pathologie, Städtisches Klinikum Braunschweig, Celler Straße 38, 38114 Braunschweig, Germany; a.dellmann@klinikum-braunschweig.de; 3 Helmholtz Center for Infection Research, Inhoffenstraße 7, 38124 Braunschweig, Germany

**Keywords:** *L. pneumophila*, ProA, FlaA, human lung tissue explants, TLR5

## Abstract

The environmental bacterium *Legionella pneumophila* is an intracellular pathogen of various protozoan hosts and able to cause Legionnaires’ disease, a severe pneumonia in humans. By encoding a wide selection of virulence factors, the infectious agent possesses several strategies to manipulate its host cells and evade immune detection. In the present study, we demonstrate that the *L. pneumophila* zinc metalloprotease ProA functions as a modulator of flagellin-mediated TLR5 stimulation and subsequent activation of the pro-inflammatory NF-κB pathway. We found ProA to be capable of directly degrading immunogenic FlaA monomers but not the polymeric form of bacterial flagella. These results indicate a role of the protease in antagonizing immune stimulation, which was further substantiated in HEK-Blue^TM^ hTLR5 Detection assays. Addition of purified proteins, bacterial suspensions of *L. pneumophila* mutant strains as well as supernatants of human lung tissue explant infection to this reporter cell line demonstrated that ProA specifically decreases the TLR5 response via FlaA degradation. Conclusively, the zinc metalloprotease ProA serves as a powerful regulator of exogenous flagellin and presumably creates an important advantage for *L. pneumophila* proliferation in mammalian hosts by promoting immune evasion.

## 1. Introduction

*Legionella pneumophila*, the causative agent of Legionnaires’ disease, was discovered in 1976 after an epidemic outbreak of atypical pneumonia at the American Legion convention in Philadelphia [1,2]. Via inhalation of *Legionella-*containing aerosols, the environmental bacterium is able to enter the human lung and replicate within macrophages or lung epithelial cells [3,4,5]. However, mammalian host cells have evolved many strategies to recognize and defend invading pathogens. The innate immune system plays an indispensable role in detecting conserved pathogen associated molecular patterns (PAMPs) with germline-encoded receptors [6,7,8]. Two key groups of these so-called pattern recognition receptors (PRRs) are the Toll-like receptors (TLRs) and the nucleotide-binding oligomerization domain (Nod)-like receptors (NLRs) [9]. TLRs detect a broad spectrum of PAMPs, mostly complex molecules consisting of lipids, carbohydrates and peptides [10].

A very specific pathway is the recognition of extracellular bacterial flagellin by membrane-bound TLR5 [11]. Flagellin is the major component of the surface-exposed flagellar filament, which is attached via the flagellar hook to the basal body [12,13,14]. The flagellin monomer consists of four globular domains (D_0_, D_1_, D_2_ and D_3_), whereby the D_1_ domain is highly conserved within both Gram-positive and Gram-negative bacteria. Especially this D_1_, but also the D_0_ polymerization domain, are crucial for TLR5 recognition [15,16,17]. TLR5 stimulation by flagellin initiates a complex signaling cascade, where the phosphorylated Interleukin-1 receptor-associated kinase-1 (IRAK-1) is released from the TLR5/MyD88 (Myeloid differentiation primary response 88) complex. IRAK-1 causes the activation of IκB kinase IKK as well as degradation of the inhibitor IκB, which, in turn, results in activation of the transcription factor NF-κB (nuclear factor kappa-light-chain-enhancer of activated B cells) and cytokine expression [18]. In *L. pneumophila*, the expression of a single monopolar flagellum is life-cycle-dependent and correlates with the virulent post-exponential growth phase. Especially the major subunit FlaA enhances the invasion capacity and mediates toxicity to host cells. Non-flagellated mutants of *L. pneumophila* are hence less infective for amoeba and macrophages [19,20,21]. However, free not-assembled FlaA induces innate immunity of alveolar macrophages and lung epithelial cells. This leads, for example, to the expression of IL-8, which attracts and activates especially polymorphonuclear neutrophils (PMNs) in inflammatory regions of the alveolar space [22,23,24,25,26]. Recognition of *L. pneumophila* flagellin by extracellular TLR5 thus plays an important role for bacterial clearance by the host immune response. A common stop-codon polymorphism in the ligand-binding domain of TLR5 even correlates with susceptibility to Legionnaires’ disease [23].

Nevertheless, pathogenic bacteria have evolved many strategies to evade host cell-mediated immune recognition, which should be addressed in this study [27,28,29]. Some pathogens developed mechanisms to prevent TLR5 activation like *Pseudomonas aeruginosa* utilizing its virulence proteases AprA and pseudolysin to attack exogenous flagellin monomers [30,31,32]. The major secretory protein ProA of *L. pneumophila* represents an M4 zinc metalloprotease highly homologous to the elastase pseudolysin [33,34,35]. The expression of ProA is, accordingly to the virulent phenotype of *L. pneumophila*, especially induced during the stationary growth phase [36]. Initially, the protease is translated as a preproenzyme and matures to a 38 kDa peptidase after release into the extracellular space via the type II secretion system [34]. It shows hemolytic activity and furthermore hydrolyzes a versatile spectrum of substrates comprising host factors like collagen IV or human serum proteins [35,37,38,39,40]. Additionally, the protease is associated with lung lesions and tissue necrosis, and can hinder different types of immune cells [41,42,43]. In a human lung tissue explant model, we earlier demonstrated how effectively *L. pneumophila* can use the lung as a habitat and lead to substantial tissue destruction [44,45,46]. In this case, ProA massively contributed to the observed damage during infections and induced a phenotype with significantly increased alveolar septal thickness [40]. A very specific function of the protease is the direct activation of the glycerophospholipid:cholesterol acyltransferase PlaC from *L. pneumophila*, leading to the suggestion that ProA also acts as a regulator in host infection processes [47].

In this study, we revealed that the zinc metalloprotease ProA is able to regulate FlaA, another bacterial substrate, and clarified its role as a specific TLR5 signaling inhibitor. Thereby, we demonstrated that *L. pneumophila*, actually known as an environmental-associated bacterium and, per se, not adapted to the human host, exerts strategies which explicitly influence mammalian host inflammation pathways.

## 2. Materials and Methods

### 2.1. Cultivation of Bacteria and Eukaryotic Cell Lines

All strains, plasmids and primers used for the experiments of this study are listed in Table 1. The *L. pneumophila* Corby wild type (WT) was cultivated on buffered charcoal yeast extract (BCYE) agar for 3–5 days at 37 °C. Liquid cultures were grown in yeast extract broth (YEB) at 200 rpm and 37 °C to the stationary phase. All media were supplemented with 20 µg/mL kanamycin (Km) or 12 µg/mL chloramphenicol (Cml) for the cultivation of *L. pneumophila* Corby mutant strains. HEK-Blue™ hTLR5 cells were cultured in DMEM with 4.5 g/L glucose, 10% (*v*/*v*) FBS, 2 mM glutamine, 30 µg/mL blasticidin and 100 µg/mL Zeocin™ at 37 °C and 5% CO_2_.

### 2.2. Isolation and Purification of Zinc Metalloprotease ProA

Native ProA production and purification were performed as recently described [40]. *L. pneumophila* Corby WT was grown overnight in 1 L YEB. Culture supernatant was obtained by centrifugation at 5000× *g* and 4 °C for 20 min, and subsequent filtration to eliminate cells. Secreted proteins were precipitated with 60% (*w*/*v*) ammonium sulfate. After incubation at 4 °C overnight, the suspension was centrifuged for 30 min at 20,000× *g* and 4 °C. The resulting protein pellet was resuspended in 20 mM Tris-HCl pH 7.5 and dialyzed against 2 L of the same buffer. ProA was purified via anion exchange chromatography (DEAE-Sepharose^®^, Sigma-Aldrich, Taufkirchen, Germany) and eluted with 20 mM Tris-HCl pH 7.5 and 1 M NaCl. Pure protease fractions (eluates 12–33) were identified by SDS-PAGE (Figure S1). They were pooled and concentrated via Amicon Ultra-15 filter centrifugal units (30,000 MWCO, Merck, Darmstadt, Germany). Additionally, ProA was rebuffered in 50 mM Tris-HCl pH 7.5 with 100 mM NaCl and 5% (*v*/*v*) glycerin for storage at −20 °C. Protein concentration was determined by Roti^®^-Nanoquant (Carl Roth, Karlsruhe, Germany) Bradford assay, and protease activity was measured by azocasein assay [51].

### 2.3. Isolation of Native Flagellin

Flagellin was isolated from *L. pneumophila* (FlaA) and *P. aeruginosa* (FliC) similar to Montie et al. [52]. The *L. pneumophila* Corby *proA* deletion mutant was cultivated in YEB overnight and plated on BCYE. After 5 d at 30 °C, the bacteria were rinsed from the agar plate with 0.01 M potassium phosphate buffer pH 7.0 and centrifuged for 15 min at 5000× *g* and 4 °C. The resulting pellet was resuspended in phosphate buffer, and the cells were homogenized for 3 min with a blender. After centrifugation at 16,000× *g* and 4 °C for 15 min, the supernatant was collected and again centrifuged at 40,000× *g* and 4 °C for 3 h. The resulting protein pellet was suspended in a small amount of 50 mM Tris-HCl pH 7.5 with 100 mM NaCl and 1% (*v*/*v*) glycerin. Protein concentration was measured via Bradford assay, purity was determined by SDS-PAGE.

### 2.4. Flagellin Degradation Assay

FlaA or FliC degradation assays were performed according to Bardoel et al. by incubating different concentrations of natively purified ProA with 10 µg/mL monomeric or polymeric flagellin diluted in PBS [31]. For depolymerization, flagella were previously heated at 70 °C for 20 min. Cleavage was analyzed after 1 h at 37 °C via a silver-stained 12% (*v*/*v*) SDS gel and immunoblotting. Following an SDS-PAGE, the proteins were transferred to a PVDF membrane by semi-dry method. After blocking for 1 h with Tris-buffered saline with 0.05% (*v*/*v*) Tween 20 (TBS-T) and 5% (*w*/*v*) milk powder, the blot was incubated with a polyclonal rabbit α-FlaA antibody overnight [53]. After washing, the membrane was incubated with the alkaline phosphatase (AP)-linked goat α-rabbit IgG (Fisher Scientific, Schwerte, Germany) secondary antibody for 1 h, followed by colorimetric protein visualization with NBT/BCIP. Relative band intensities of FlaA in stained SDS gels were determined using ImageJ [54].

### 2.5. Collection of Human Lung Tissue Explant Supernatant

Tumor-free human lung tissue explants (HLTEs) were obtained from surgery patients and infected as described previously [44,45,46]. RPMI 1640 with 10% (*v*/*v*) FBS, 20 mM HEPES and 1 mM sodium pyruvate was inoculated with *L. pneumophila* Corby from stationary phase cultures at a density of 10^7^ bacteria/mL. The vital lung specimen was cut into pieces of 0.1 g and transferred into the bacterial suspension. Tissue samples were incubated up to 24 h at 37 °C and 5% CO_2_ in a 24-well format. For inflammatory response analysis, supernatant was isolated at indicated time points and stored at −80 °C until measurement.

### 2.6. HEK-Blue™ hTLR5 Detection Assay

HEK-Blue™ hTLR5 cells (InvivoGen) were seeded in 96-well cell culture plates 24 h prior to stimulation at a density of 2.52 × 10^4^ cells/well in cell culture medium. Before adding the proteins, bacteria or infection supernatant samples, cells were washed once with sterile medium. From each sample and negative control, 20 µL were applied. In bacterial approaches, stationary cultures adjusted to 10^8^ CFU/mL were used. Protein samples were derived from the native source and used in final concentrations of 0.05–100 ng/mL for *L. pneumophila* FlaA and 0.1–5000 ng/mL for ProA. After adding 180 µL of HEK-Blue™ Detection medium (InvivoGen), the eukaryotic cells were incubated for 16 h at 37 °C and 5% CO_2_. The cell line contains an SEAP (secreted embryonic alkaline phosphatase) reporter gene, which is induced by the transcription factors NF-κB and AP-1 in the downstream signaling process after receptor stimulation of hTLR5. SEAP activity was measured at OD_620_.

### 2.7. Statistical Analyses

All experiments were carried out in duplicates and reproduced at least three times. Statistical analyses were performed using a one-way ANOVA for repeated measurements and Dunnett’s post hoc test for simple effect analyses in GraphPad Prism version 8.2.0 for Windows, GraphPad Software, San Diego, California, CA, USA, www.graphpad.com. Beforehand, normal distribution of the data was reviewed via Shapiro-Wilk test, and residual analyses were graphically evaluated via QQ plots. Differences were considered significant at *p* ≤ 0.05.

## 3. Results

### 3.1. Zinc Metalloprotease ProA Degrades Monomeric Flagellin

**Figure 1:**
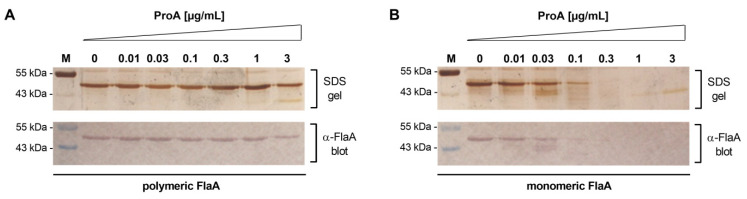
ProA degrades monomeric flagellin. Flagella were isolated from *L. pneumophila* Corby and heated for 20 min to 70 °C for depolymerization. Polymeric (**A**) and monomeric (**B**) flagellin (10 µg/mL) was incubated for 1 h at 37 °C with indicated concentrations of purified ProA. Samples were separated in an SDS gel with subsequent protein silver staining. M: 1–5 µL of the PageRuler^TM^ Prestained Protein Ladder by Thermo Scientific were used as a standard. Additionally, the samples were immunoblotted with a primary antibody against *L. pneumophila* FlaA and detected with an alkaline phosphatase antibody and NBT/BCIP substrate solution. While the polymeric form of flagellin is resistant against the proteolytic degradation by ProA, FlaA monomers were efficiently cleaved by the protease. Not more than one third of the initial substrate concentration was left after inoculation of FlaA monomers with 0.1 µg/mL ProA.

In a previous study, Casilag and colleagues presented that the elastase pseudolysin from *P. aeruginosa*, a ProA homologue of the same enzyme family, prevents flagellin-mediated immune recognition via cleavage of flagella subunits [32,55]. To analyze if *L. pneumophila* ProA is also able to degrade flagellin*,* the purified protease was incubated in defined concentrations with polymeric (complete flagellar filaments) and monomeric forms of FlaA (Figure 1). An SDS-PAGE as well as Western blot analyses with specific immunostaining showed that 10 µg/mL polymeric flagellin is even stable during co-incubation with 3 µg/mL of ProA (Figure 1A). In contrast, monomeric FlaA was efficiently cleaved by small amounts of the *L. pneumophila* protease (Figure 1B). Relative band intensities from three independent experiments supported stability of FlaA polymers as well as gradual digestion of monomers. It revealed that monomeric flagellin inoculated with 0.1 µg/mL of ProA was reduced to one third of the initial concentration after 1 h, while polymeric FlaA was still stable to ten times more ProA (Figure S2). These results demonstrated that ProA only degrades depolymerized flagellin and not entire filaments, which retains the functionality of bacterial flagella. Control experiments with heat-inactivated protease additionally verified that observed FlaA cleavage is directly dependent on the ProA enzyme activity (Figure S3).

Moreover, similar degradation experiments with the filament subunit FliC from *P. aeruginosa* and the *L. pneumophila* protease indicate a rather conserved cleavage site of the flagellin molecules, which enables ProA to even recognize monomers of other bacterial species (Figure S4). Our observations were strongly supported by the fact that depolymerization of samples isolated from the wild type of *L. pneumophila* or *P. aeruginosa* led to a decisive loss of flagellin. This is most likely due to residual protease activity after purification, which is only able to degrade monomeric but not polymeric forms. Consistently, FlaA purified from the *proA*-deficient *L. pneumophila* strain was not altered during the process of monomerization and was therefore used in this study (Figure S5).

### 3.2. Flagellin-Mediated TLR5 Activation Is Prevented by ProA

**Figure 2:**
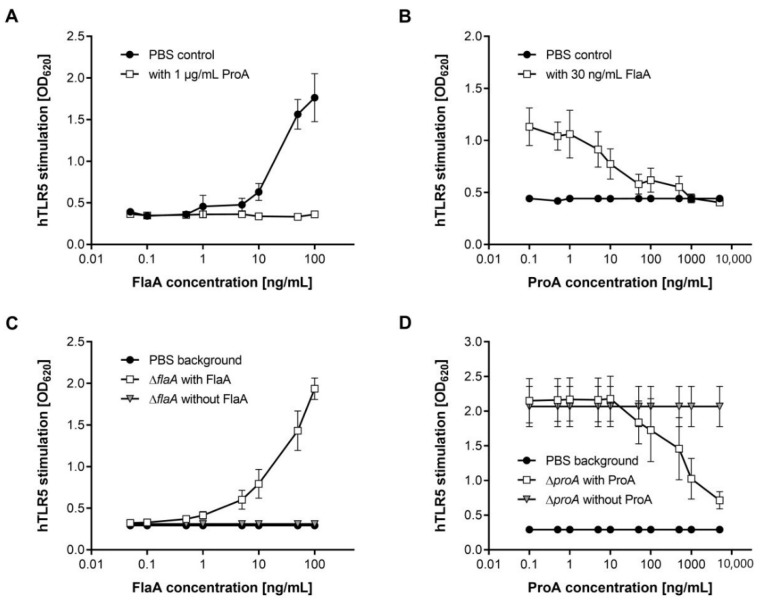
ProA inhibits TLR5 activation by FlaA in HEK-Blue™ cells. HEK-Blue™ hTLR5 cells were seeded at a density of 2.52 × 10^4^ cells/well. After adherence, they were inoculated with HEK-Blue™ Detection medium and 20 µL samples containing the indicated bacterial strains or proteins. PBS served as a negative or background control (black dots). After incubation at 37 °C and 5% CO_2_ for 16 h, SEAP activity, and hence hTLR5 stimulation, were determined at OD_620_. Means of measurements are shown with ±SEM from three (**A**,**B**) or four (**C**,**D**) independent experiments. (**A**) HEK-Blue™ cells were treated with PBS or 1 µg/mL ProA (white squares) and different concentrations of purified FlaA, only leading to increasing hTLR5 activation in the negative control. (**B**) 30 ng/mL FlaA per well (white squares) were added to HEK-Blue™ cells, which were additionally treated with different ProA concentrations between 0.1 ng/mL and 5000 ng/mL. TLR5 stimulation was thereby inhibited in a concentration-dependent manner. (**C**) HEK-Blue™ cells were inoculated with the *L. pneumophila* Corby Δ*flaA* mutant (grey triangles). Co-incubation with purified flagellin reconstituted the lacking TLR5 stimulation of the eukaryotic cells (white squares). (**D**) *L. pneumophila* Corby Δ*proA* inoculation of HEK-Blue™ cells resulted in high hTLR5 activation (grey triangles), but was diminished by adding natively purified ProA in increasing concentrations (white squares).

Bacterial flagellin is a potent inducer of pro-inflammatory host responses and is extracellularly detected by TLR5 on host cell surfaces [11,56]. To elucidate if ProA-dependent FlaA degradation alters the innate immune recognition by mammalian cells, we performed a specific detection assay with the hTLR5-expressing cell line HEK-Blue™. In substrate-containing medium, SEAP reporter gene activity induced by NF-κB signaling after TLR5 activation was measured at OD_620_. Co-incubation of *L. pneumophila* flagellin with HEK-Blue™ cells accordingly showed concentration-dependent hTLR5 stimulation (Figure 2A). The more FlaA was added, the more SEAP was expressed, and thus its colorimetric product led to higher absorbance at OD_620_.

Interestingly, such TLR5 activation was completely inhibited by adding 1 µg/mL of purified ProA to the experimental setup. Even at concentrations of 100 ng/mL FlaA, no TLR5 activation was observed in the presence of ProA. This was directly dependent on the proteolytic activity, since a control reaction with heat-inactivated protease was not able to reduce TLR5 stimulation by purified *L. pneumophila* flagellin. It was additionally validated that ProA does not exhibit activity against host proteins like TLR5 on the cell surface, and observed effects are thus due to FlaA cleavage (Figure S6). Different doses of ProA were tested in the HEK-Blue™ Detection assay to determine which concentration of the *Legionella* protease is sufficient for preventing TLR5 detection of 30 ng/mL FlaA (Figure 2B). The reduction of OD_620_ values correlated with increasing ProA concentrations, and TLR5 stimulation was again abolished by using 1 µg/mL of ProA. These data clearly demonstrate that *L. pneumophila* ProA is able to diminish TLR5 activation by FlaA effectively.

To further analyze these effects, hTLR5 on HEK-Blue™ cells was activated by whole bacterial cells. Co-incubation with a *L. pneumophila* Corby *flaA* deletion mutant only resulted in SEAP activity similar to the negative PBS control (Figure 2C). By additionally adding purified FlaA in different concentrations, the TLR5 stimulation was clearly increased. Consequently, downstream pathways seem not to be unspecifically triggered by other bacterial factors besides flagellin. In contrast, a *proA*-negative mutant immediately induced high TLR5 response as measured by absorbance at OD_620_ after co-inoculation with HEK-Blue™ cells (Figure 2D). Reduction of the observed TLR5 activation was provoked by the addition of at least 50 ng/mL of purified ProA. With higher protease concentrations, the SEAP activity decreased in a concentration-dependent manner of ProA. An addition of 10 µg/mL of purified protease presumably abolishes the TLR5 activation induced by a *proA* deletion mutant. All these data demonstrate that extracellular ProA, which is also secreted by *L. pneumophila* during infections*,* is able to decrease TLR5 activation by cleavage of free monomeric flagellin in the surrounding medium of bacterial cells.

### 3.3. Immune Recognition of the L. pneumophila WT via TLR5 Is Reduced by ProA

**Figure 3:**
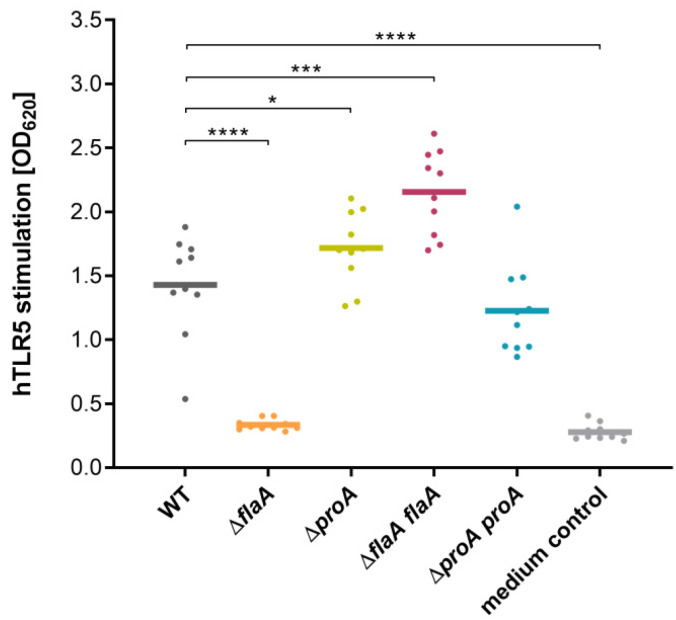
Specific TLR5 activation in HEK-Blue™ cells by different *L. pneumophila* strains. HEK-Blue™ hTLR5 cells at a density of 2.52 × 10^4^ cells/well were incubated with 180 µL detection medium and 20 µL culture of the *L. pneumophila* Corby wild type (WT), the negative mutant strains Δ*proA* and Δ*flaA* or complementants Δ*proA proA* and Δ*flaA flaA* in a 96-well format. Prior to inoculation, bacterial strains were grown to stationary phase in YEB, and the cell count was adjusted to 10^8^ CFU/mL. After incubation of the assay plate at 37 °C and 5% CO_2_ for 16 h, TLR5-dependent NF-κB activation was measured by SEAP activity at OD_620_. The results are depicted in a scatter plot with means and reveal significantly diminished values in the case of the *flaA*-negative strain but even stronger induced TLR5 stimulation by the *proA* deletion mutant compared to the WT. Complementation strains of Δ*proA* and Δ*flaA* were able to completely reconstitute these phenotypes. Statistics were performed using a repeated measurement one-way ANOVA with Dunnett’s post hoc test for simple effect analysis. Significant results of ten biological replicates are indicated with asterisks (* *p* ≤ 0.05, *** *p* ≤ 0.001, **** *p* ≤ 0.0001).

In further bacterial analyses, the HEK-Blue™ Detection assay was performed to directly differentiate the TLR5 responses evoked by the *L. pneumophila* Corby WT compared to a *proA*-negative and a *flaA-*negative mutant, as well as the functional complementation strains ∆*proA proA* and ∆*flaA flaA* (Figure 3). Measurements with 10^7^ bacterial CFU/mL revealed OD_620_ values of 1.4 for the *Legionella* WT. ProA deletion resulted in a significant increase of TLR5 activation to an OD_620_ of 1.7, whereas stimulation by the *flaA*-deficient mutant is almost abolished to the background level of the medium control. Additionally, these phenotypes were fully restored by using the corresponding complemented strains. FlaA overexpression in ∆*flaA* even led to enhanced TLR5 stimulation compared to the wild type. Altogether, these results demonstrate a repression of *L. pneumophila* flagellin-induced TLR5 activation by ProA, which is also detectable in comparison of ProA-expressing or non-expressing bacterial strains.

### 3.4. ProA Diminishes Extracellular FlaA Stimuli during Human Lung Tissue Infections

**Figure 4:**
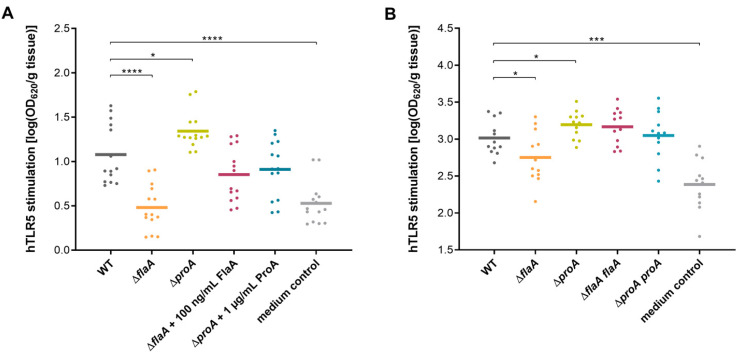
TLR5 activation in HEK-Blue™ cells by supernatants of infected lung tissue explants (HLTEs). Explanted human lung tissue specimens were infected with the *L. pneumophila* Corby WT, a *proA*-negative mutant Δ*proA* and a *flaA*-negative mutant Δ*flaA*. Loss of ProA or FlaA was reconstituted either by addition of purified proteins (1 µg/mL ProA and 100 ng/mL monomeric FlaA) (**A**) or use of the specific complementation strains Δ*proA proA* and Δ*flaA flaA* (**B**). Untreated tissue samples served as a negative control. Infected tissue pieces were weighed, and supernatants were isolated and incubated with HEK-Blue™ hTLR5 cells for 16 h at 37 °C and 5% CO_2_. Activation of the TLR5-NF-κB pathway was measured by SEAP activity at OD_620_ and is shown in scatter plots with means. Compared to the *L. pneumophila* WT, a HEK-Blue™ Detection assay revealed significantly reduced TLR5 activation by Δ*flaA*-infected tissue supernatant and elevated stimulation with the Δ*proA* mutant strain. These effects were apparent after 2 h (**A**) and 24 h (**B**) post infection and were successfully restored by complementation with respective proteins or gene sequences. Significance of at least twelve replicates was evaluated by repeated measurement one-way ANOVA with Dunnett’s post hoc test for simple effect analysis (* *p* ≤ 0.05, *** *p* ≤ 0.001, **** *p* ≤ 0.0001).

For analyzing the ability of *L. pneumophila* to evade the TLR5-dependent immune response on tissue level, we infected human lung tissue explants (HLTEs) with different *Legionella* mutant strains. This model was previously demonstrated to be capable of revealing intra- and extracellular aspects of the host–pathogen interaction during Legionnaires’ disease via bacterial growth kinetics, transmigration and localization as well as histopathological alterations of the lung tissue architecture [44,45,46]. Therefore, it is well suitable to compare pro-inflammatory host cell responses in infections with the wild type (WT), a *proA*- and a *flaA-*negative mutant (Figure 4). Furthermore, potential phenotypes were reconstituted by the addition of purified proteins (30 ng/mL FlaA and 1 µg/mL ProA) or the functional complementation strains ∆*proA proA* and ∆*flaA flaA*. Infected human lung tissue pieces were incubated for different time intervals at 37 °C in cell culture medium. To investigate TLR5 stimulation in the different samples, HEK-Blue™ cells were co-incubated with HLTE infection supernatants from 2 h (Figure 4A) or 24 h (Figure 4B) post inoculation.

The results showed significant differences in the hTLR5 activation potential of all used *Legionella* deletion strains. At both time points, infection with a *proA*-negative mutant resulted in significantly higher TLR5 activation than with the wild type. In contrast to that, tissue supernatant of a *flaA*-deficient mutant strain showed lower OD_620_ values comparable to the medium control. By the addition of purified ProA, the *L. pneumophila* Δ*proA* phenotype could be restored. The enhanced immune response stimulation of this deletion mutant was also significantly reduced using the Δ*proA proA* complementant. Equivalently, reconstitution was observed for co-incubation of HLTEs with the *flaA*-deficient mutant and corresponding protein supplementation or complementation strain samples. Our results demonstrate that *L. pneumophila* Corby flagellin triggers TLR5-dependent immune response during infections of human lung tissue, which can be significantly reduced by expression of ProA. Interestingly, this effect seems to be most important during the first hours of infection, whereas an established infection generally leads to a stronger activation of the NF-κB pathway in exposed human cells.

## 4. Discussion

In this study, we were able to identify *L. pneumophila* flagellin FlaA as a physiological substrate of the bacterial zinc metalloprotease ProA. Interestingly, the enzyme only targets the immunogenic monomeric form and leaves polymerized FlaA intact. Our immunological experiments clarified the role of ProA as a regulator of another bacterial virulence factor. In HEK-Blue^TM^ Detection assays, the FlaA-mediated stimulation of TLR5 exposed on host cells was measured with purified proteins, bacterial suspensions of deletion and complementation mutants but also with supernatants of infected HLTEs. Receptor stimulation by a *flaA*-deficient mutant equated to the medium control and was significantly reduced compared to the *L. pneumophila* WT or other FlaA-containing samples. Furthermore, the absorbance at 620 nm due to exogenous flagellin was decreased by supplementation with the protease ProA. Bacterial suspensions or infection supernatants containing a *proA* deletion strain showed an increased TLR5 activation, indicating an important role of the zinc metalloprotease in counteracting pro-inflammatory properties of bacterial flagellin. This supports the notion that recognition of FlaA and subsequent TLR5-mediated immune response plays an important role for pathogen clearance during human *Legionella* infections. Patients with a TLR5^392STOP^ polymorphism in the ligand-binding domain, accordingly, exhibit an increased susceptibility for Legionnaires’ disease [23].

Since the TLR5 receptor as well as the protease ProA exclusively target monomeric flagellin, in both cases, the recognition sites are most likely hidden inside the polymerized flagellum. In this context, it was already published that amino acid residues of the conserved D_1_ domain, but also the flagellin polymerization domain D_0_, are specifically involved in TLR5 binding [16,17]. This would allow ProA to induce immune inhibitory effects without influencing bacterial motility. Similar roles were already reported for the homologous M4 protease pseudolysin as well as AprA of *P. aeruginosa*, which reduces flagellin-mediated TLR5 activation completely. Thus, an *aprA*-negative mutant causes a 100-fold enhancement in receptor stimulation compared to the WT, even exceeding the deletion effect of ProA in *L. pneumophila* Corby [31,32].

**Figure 5:**
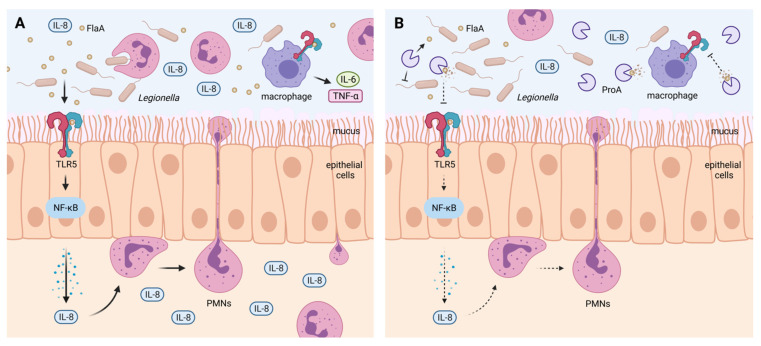
Proposed mechanism of counteracting flagellin-mediated host immune response by the *L. pneumophila* protease ProA. (**A**) During lung infection with *L. pneumophila*, bacterial FlaA monomers are recognized via TLR5 presented on the surface of lung epithelial cells or alveolar macrophages. Stimulation of the receptor initiates a signaling cascade which results in NF-κB activation and IL-8 production. IL-8 is a main chemoattractant of PMNs triggering recruitment into the infected region and subsequent pathogen clearance. Alveolar macrophages additionally express pro-inflammatory cytokines like IL-6 and TNF-α upon TLR5 stimulation. (**B**) *L. pneumophila* secretes the zinc metalloprotease ProA which is able to cleave immunogenic FlaA monomers, while the intact flagellum, and hence bacterial motility, are not affected by its proteolytic activity. Efficient degradation of monomeric flagellin results in a strong reduction of TLR5 activation and the following cytokine production. Consequently, stimulation, recruitment and bacterial killing of PMNs will be reduced. Thus, ProA may function as an important modulator of the FlaA-mediated pro-inflammatory host response (created with BioRender.com).

TLR5 is predominantly expressed on alveolar macrophages but also on type II pneumocytes, epithelial, endothelial and dendritic cells. A decreasing TLR5 activation alters expression and secretion of chemokines as a part of the pro-inflammatory immune response [57,58]. Via the NF-κB pathway, TLR5-mediated recognition of FlaA provokes, for example, the production of TNF-α, which plays a protective role during *L. pneumophila* infections. This important immune mediator induces apoptosis of infected host cells, hence reducing proliferation and bacterial load in the lung [24,59]. In this context, it was already shown that *L. pneumophila* ProA is able to dampen the chemokine and cytokine output from infected macrophages [60]. The protease also exhibits direct proteolytic activity against TNF-α, and the interleukins IL-2 and IL-6 [60,61,62]. Moreover, mice experiments with *P. aeruginosa* additionally revealed a TLR5-dependent expression of the chemokines MIP-2, IL-17, and IL-22 as well as antimicrobial peptides such as β-defensin 2 or CRAMP (*cathelin-dependent antimicrobial peptide*) [63]. Among different chemokines, IL-8 is one of the most important pro-inflammatory host messengers, which is expressed in a TLR5-dependent, and thus also in a FlaA-dependent, manner [64,65,66]. It participates in the recruitment of immune cells, especially PMNs, for the clearance of the pathogen [24,67]. Moreover, IL-8 was the cytokine with the highest level in culture supernatants of *L. pneumophila*-infected macrophages [60]. Results of this study point to a reduced secretion of pro-inflammatory host factors like IL-8 and TNF-α due to a repression of the TLR5-NF-κB pathway by ProA-expressing *Legionella* strains (Figure 5). We therefore hypothesize that the zinc metalloprotease is able to diminish the IL-8-dependent recruitment and cellular immune response of PMNs during human lung tissue infection.

Altogether, our results indicate that cleavage of free flagellin by ProA promotes immune evasion and, in turn, might represent an important advantage for replication of the pathogen in the human host. Moreover, the immune inhibitory function of the protease is not affecting the polymerized flagellum of the bacterium, and thus its role during the proliferation inside pulmonary lung tissue. Conclusively, we revealed a new component and mechanism of the extensive spectrum of targets by which ProA contributes to different aspects of *L. pneumophila* pathogenesis. Besides the extracellular detection of FlaA by TLR5 on host cells, flagellin is also sensed in the cytosol by the Naip5/Nlrc4 inflammasome [68]. It is likely that ProA is also able to degrade flagellin, which is inadvertently translocated into the host cell depending on the *L. pneumophila* dot/icm type IV secretion system (T4SS), since the protease was previously found to assemble at the cytoplasmic site of the LCV. Similar to FlaA, the zinc metalloprotease might be translocated into the host cell cytoplasm due to unspecific pore formation by the T4SS or another bacterial factor of the LCV lumen. However, it is also possible that the semipermeability of the LCV results from host cell membrane transporters, which are acquired via ER-derived vesicles during the first hours after bacterial invasion [69,70,71]. Hence, in the future, it will be particularly interesting to investigate to which extent ProA might additionally influence the intracellular activation of a FlaA-mediated immune response.

## Figures and Tables

**Table 1:** List of bacterial strains, cell lines, plasmids and primers.

**Cell Line**	**Description**	**References**
HEK-Blue^TM^ hTLR5	HEK 293 cell line expressing human TLR5, SEAP reporter	InvivoGen, Toulouse, France
**Strain**	**Description**	**References**
*L. pneumophila* Corby WT	WT reference strain	[48]
*L. pneumophila* Corby Δ*proA*	*proA::nptI*, *proA* deletion mutant, Km^r^	[40]
*L. pneumophila* Corby Δ*proA proA*	*proA* deletion mutant with pMMB2002-*proA*, Km^r^, Cml^r^	[40]
*L. pneumophila* Corby Δ*flaA*	*flaA::nptI*, *flaA* deletion mutant, Km^r^	[49]
*L. pneumophila* Corby Δ*flaA flaA*	*flaA* deletion mutant with pMMB2002-*flaA*, Km^r^, Cml^r^	This study
*P. aeruginosa* PAO1	WT strain	ATCC 15692
**Plasmid**	**Description**	**References**
pMMB2002	pMMB207-derived expression plasmid for *L. pneumophila*, deleted *mobA* gene, Cml*^r^*	[50]
pMMB2002-*proA*	pMMB2002-derived vector expressing the *proA* gene, Cml*^r^*	[40]
pMMB2002-*flaA*	pMMB2002-derived vector expressing the *flaA* gene, Cml*^r^*	This study
**Primer**	**5′-3′-Sequence**	**References**
FlaA-Pr_fw	CATGAGCTCTCGACTTGATAACCCGAACC	This study
FlaA+_rv	AAGGTACCCTATCGACCTAACAATGATAATAC	This study

## Data Availability

The data that support the findings of this study are contained within the article or Supplementary Materials.

## Supplementary Materials

The following supporting information can be downloaded at: https://www.mdpi.com/article/10.3390/biom12050624/s1, Figure S1: SDS gels of the ProA purification via anion exchange chromatography; Figure S2: Relative amounts of monomeric and polymeric FlaA in degradation assays with the zinc metalloprotease ProA from *L. pneumophila*; Figure S3: SDS gel of FlaA degradation experiments with inactive ProA; Figure S4: SDS gels of degradation experiments with FliC from *P. aeruginosa* and *L. pneumophila* ProA; Figure S5: SDS gel of flagellin polymers and monomers purified from the *L. pneumophila* WT, the deletion mutant ∆*proA* and *P. aeruginosa*; Figure S6: HEK-Blue^TM^ hTLR5 Detection assay with purified *L. pneumophila* FlaA and ProA.
