# Supplementary material for: Zinc Metalloprotease ProA from Legionella pneumophila Inhibits the Pro-Inflammatory Host Response by Degradation of Bacterial Flagellin
Source: Biomolecules. 2022 Apr 22;12(5):624. doi: 10.3390/biom12050624 (PMC9138289; doi:10.3390/biom12050624)
Supplement: Supplementary file 1 [file biomolecules-12-00624-s001.zip › biomolecules-1663470-supplementary.pdf]

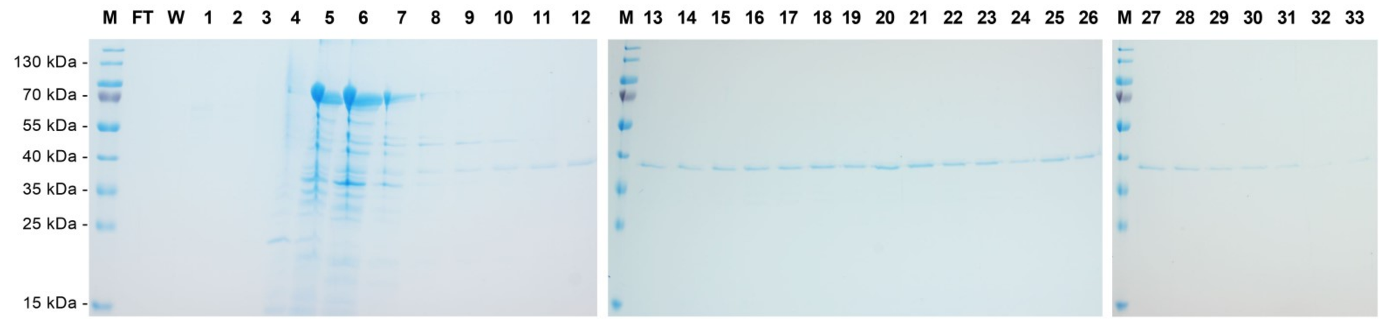

Figure S1. SDS gels of the ProA purification via anion exchange chromatography. Proteins from 1 L *L. pneumophila* culture supernatant were precipitated with ammonium sulfate, dialysed and purified via a 10 mL DEAE-Sepharose™ column. After addition of loading dye, 20 µL of the 2 mL fractions (flowthrough = FT, washing = W and eluates 1-26) and 5 µL of the PageRuler™ Prestained Protein Ladder (M) were separated in 12% SDS gels. Staining was performed with Coomassie Silver Blue. Especially the early eluates 5-7 showed a lot of different protein bands but also the specific native ProA band at 38 kDa. The late eluates 12-33 solely contained the protein of interest, and were therefore pooled and concentrated for further experiments.

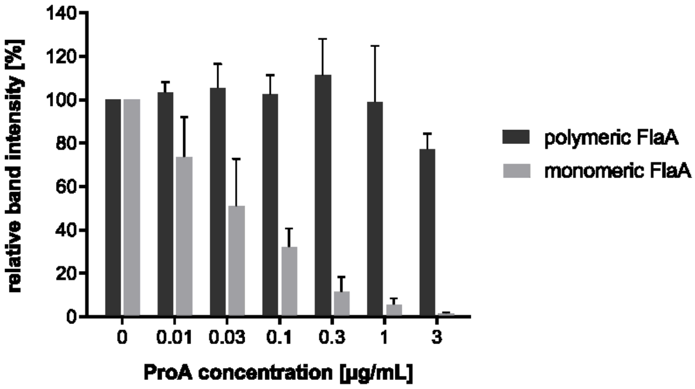

Figure S2. Relative amounts of monomeric and polymeric FlaA in degradation assays with the zinc metalloprotease ProA from *L. pneumophila*. Reactions with 10 µg/mL of flagellin and different concentrations of ProA were incubated at 37 °C for 1 h. After separation of the samples via SDS-PAGE, proteins were stained with silver nitrate. Digestions of polymers and monomers were always analysed on different gels. Band intensities were measured using the software ImageJ. Relative intensities in percentage of the initial concentration are depicted in a bar graph with standard deviation of three independent experiments. While polymeric FlaA is stable to proteolytic digestion, FlaA monomers are gradually reduced using increasing concentrations of ProA. Only one third of the initial amount is left using 0.1 µg/mL protease with 10 µg/mL substrate.

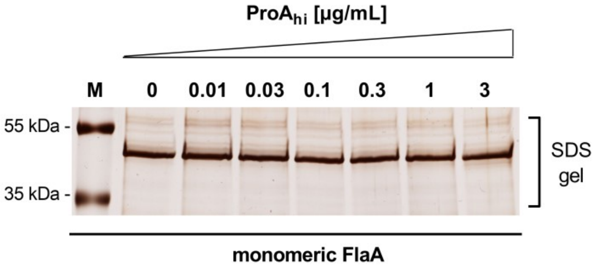

Figure S3. SDS gel of FlaA degradation experiments with inactive ProA. Both proteins were isolated from *L. pneumophila* Corby. Monomeric flagellin was generated by heat-treatment at 70 °C

for 20 min. FlaA (10 µg/mL) was incubated for 1 h at 37 °C with indicated concentrations of the heat-inactivated protease ProA. Samples were separated in an SDS-PAGE and detected via silver staining. M: 1 µL PageRuler™ Prestained Protein Ladder by Thermo Scientific was used as a standard. The inactivated variant of ProA was not able to cleave FlaA monomers.

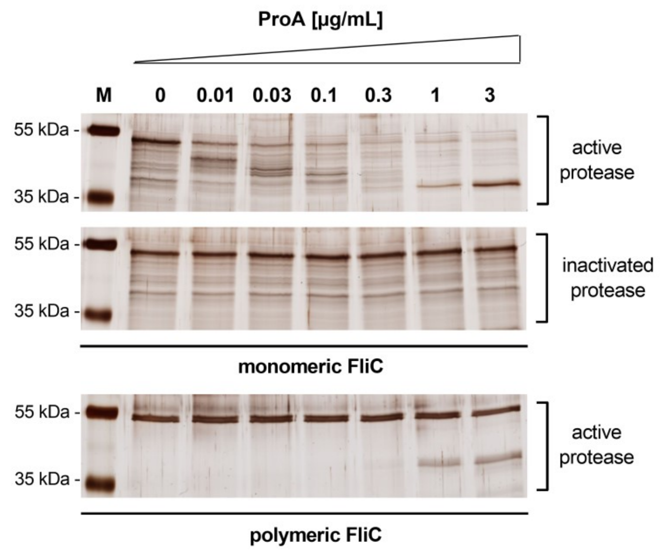

Figure S4. SDS gels of degradation experiments with FliC from *P. aeruginosa* and *L. pneumophila* ProA. Flagella were isolated from *P. aeruginosa* WT and depolymerized at 70 °C for 20 min. Polymeric (1 µg/mL) and monomeric (10 µg/mL) flagellin was incubated for 1 h at 37 °C with indicated concentrations of active or heat-inactivated protease ProA. Reactions were stopped with loading buffer, and the samples were separated in an SDS gel (12%) with subsequent protein silver staining. M: 1 µL of the PageRuler™ Prestained Protein Ladder by Thermo Scientific was used as a standard. While the polymeric form of flagellin is resistant against the proteolytic degradation by ProA, FliC monomers are efficiently cleaved by the active protease.

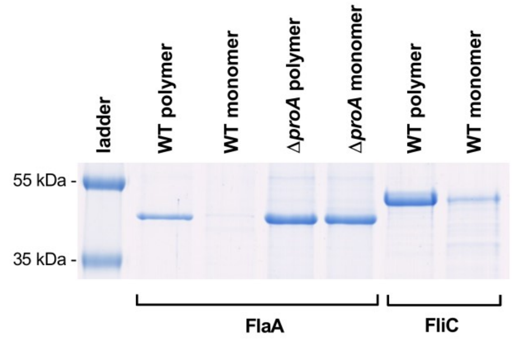

Figure S5. SDS gel of flagellin polymers and monomers purified from the *L. pneumophila* WT, the deletion mutant Δ*proA* and *P. aeruginosa*. Flagella were isolated from the bacteria via shearing and monomerized at 70 °C for 20 min. 5 µL of the PageRuler™ Prestained Protein Ladder by Thermo Scientific were used as a protein standard. 1 µg of each sample was loaded on a 12% SDS gel. Staining was performed with Coomassie Silver Blue. Flagellins purified from both wild type species showed a significant decrease regarding their band intensities after monomerization, whereas FlaA from the protease deletion mutant did not undergo a visible change.

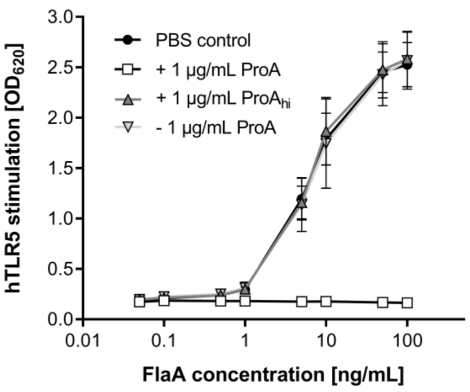

Figure S6. HEK-Blue™ hTLR5 Detection assay with purified *L. pneumophila* FlaA and ProA. HEK-Blue™ hTLR5 cells were seeded at a density of 2.52 x 10^4 cells/well prior to inoculation. After adherence for 24 h, 180 µL Detection medium and 20 µL of protein samples were added. The hTLR5 stimulation was measured via SEAP activity at OD_{620} after an incubation period of 16 h at 37 °C and 5 % CO_2. Means with ± SEM from four independent experiments are displayed. HEK-Blue™ cells were treated with different concentrations of purified monomeric FlaA. Test conditions with additionally 1 µg/mL of heat-inactivated ($ProA_{hi}$ dark grey triangles) or washed out ProA (light grey triangles) showed highly comparable results to the PBS control (black dots). Samples with 1 µg/mL active protease, however, totally abolished the signal at OD_{620}. This can only be explained by direct ProA activity against FlaA.
